# Facilitators and barriers to the implementation of Community-Based Medication Adherence Support for Aging Individuals with HIV and Hypertension in western Kenya

**DOI:** 10.21203/rs.3.rs-6897280/v1

**Published:** 2025-06-30

**Authors:** Jepchirchir Kiplagat, Violet Naanyu, Ruth Nehema, Henry Zakumumpa, Kara Wools-Kaloustian

**Affiliations:** Moi University; Academic Model Providing Access to Healthcare (AMPATH); Academic Model Providing Access to Healthcare (AMPATH); Makerere University; Academic Model Providing Access to Healthcare (AMPATH)

## Abstract

**Background:**

The advent of antiretroviral therapy (ART) remarkably improved the longevity and quality of life of people living with HIV (PLWH). However, as PLWH age, they often experience comorbidities, necessitating multiple medications, resulting in increased medication adherence challenges. Patient-tailored community-based medication adherence programs can improve adherence in this population. We explored facilitators of and barriers to the implementation of community health volunteer (CHV)-led medication adherence (CBA) support programs for older people living with HIV (OALWH).

**Methods:**

This qualitative study involved 166 purposefully sampled participants. In-depth interviews (IDIs) were held with 27 healthcare providers (HCPs), 28 CHVs, and 25 older adults’ caregivers. Six focus group discussions (FGDs) were held with 86 OALWH affiliated with three health facilities in western Kenya. The IDIs and FGDs covered topics on perceived barriers and facilitators to having a CHV visit OALWH’s home to offer medication adherence support. The data were analyzed thematically and organized using the Consolidated Framework for Implementation Research (CFIR).

**Results:**

The findings revealed various factors that could influence the implementation of a CBA intervention for OALWH and hypertension. Facilitators included the relative advantage and adaptability of the intervention, the enhanced collaboration between facility and community care providers, and the potential to promote patient-centered care. However, participants voiced several factors that may impede the intervention, such as the complexity of the intervention, increased workload and costs for CHVs, limited knowledge of hypertension management, unmet patient needs, and limited health financing for NCD medications. Fears of decreased cognitive ability, low cardiovascular risk perception, and medication side effects among OALWH were perceived to pose challenges. Furthermore, trust and empathy between CHVs and patients were identified as critical personal attributes that foster patient empowerment.

**Conclusion:**

This study identified barriers, highlighting the complexities of tailoring community support services to the needs of OALWH. The findings underscore the necessity for a holistic, multidimensional approach to addressing medication adherence by providing OALWH with the requisite hypertension management messaging, revisiting health system barriers (NCD care financing), and facilitating CHVs with knowledge, skills, and remuneration to enable them to efficiently support CBA intervention.

## Background

Globally, healthcare systems are experiencing significant change, driven by medical advancements that have greatly increased the life expectancy of people living with HIV (PLHIV) [1, 2]. Advances in HIV care and treatment have accompanied an increase in the prevalence of multimorbidity, i.e., the coexistence of two or more chronic health conditions within one individual [3–6]. These intertwined developments of chronic diseases in the HIV population have given rise to a new and complex challenge: the management of medication adherence for multiple chronic conditions in PLHIV, particularly those who are older than 50 years of age.

Adherence to medication regimens is the cornerstone of effective management of chronic diseases. For aging PLHIV with an added burden of multiple comorbidities, adherence is not only crucial but also particularly complex. The confluence of aging, HIV, and multimorbidity presents a complex healthcare scenario in which individuals must navigate a challenging network of treatment regimens, potential drug toxicities and interactions, medical appointments, lifestyle adjustments, and psychosocial factors [7–14]. Adherence support for older adults living with HIV (OALWH) and comorbid chronic diseases targeted at the above barriers may help improve their quality of life and extend their lifespan.

Task shifting in healthcare has been advocated as one of the strategies for alleviating the shortage of and workload for healthcare providers [15]. Community health volunteers (CHVs), also known as community health promoters (CHPs), are nonclinical frontline healthcare workers who have been successfully integrated into community-based health systems [16–18]. CHVs, who are based on the communities they serve, undergo relatively little training in the specific health areas that they support [19, 20]. The delivery of health services in the community offers several advantages. These include being able to contextualize health information and materials, enhancing cultural relevance, encouraging goodwill due to established trust, and working within established communication networks within the community. CHVs can also tailor healthcare services to suit community needs, and this workforce alternative is relatively cost-effective in providing care [21, 22]. While CHVs may face several challenges, such as the complex nature of tasks like disease diagnosis and the need for regular supportive supervision and training, evidence for their role in alleviating workforce shortages in resource-limited settings is accumulating [23, 24]. Despite their potential to benefit overall patient health and well-being, many healthcare models underutilize CHVs. The use of CHVs to support medication adherence for OALWH and other chronic conditions can increase adherence rates while fostering stronger collaboration among healthcare providers, community care systems, and patients.

The Academic Model Providing Access to Healthcare (AMPATH) is a large HIV treatment program in western Kenya that serves more than 127,000 people living with HIV [25, 26]. Despite the program’s ability to improve access to HIV care, preliminary studies revealed challenges faced by OALWH, including limited time for detailed discussions with clinicians due to high patient volumes [12]. OALWH who have additional chronic diseases often navigate multiple providers with limited care coordination, resulting in increased pill burden and a higher risk of drug‒drug interactions that may affect adherence and overall health [27]. HIV-related stigma and limited social support further challenge adherence for OALWH [28]. Additionally, logistical barriers such as distance to clinics and the need for accompaniment during visits are barriers to medication adherence.

Given these complexities, CHVs have emerged as potentially valuable resources for supporting medication adherence and providing basic education for OALWH in community settings. The community-based medication adherence support model (CBA), led by CHVs, ideally consists of the following proposed core components: regular home visits, blood pressure (BP) measurements, data recording, medication arrangement in a pill box, psychosocial support, health education, and teleconsultations and referrals where necessary. In this study, we sought to explore the facilitators of and barriers to the successful implementation of a CBA intervention for OALWH with hypertension in western Kenya.

## Methods

### Study Design

We adopted a qualitative exploratory research design [29]. We aimed to understand the perceived facilitators of and barriers to the implementation of a CVH-led CBA intervention from the perspective of healthcare providers, OALWH, community members, and CHVs. We also sought to document their preferences and suggestions for improving the proposed CBA intervention.

#### Study setting

This project was conducted in three facilities within the AMPATH program and their catchment areas in western Kenya. The AMPATH program provides comprehensive HIV care services in more than 500 Ministry of Health facilities in western Kenya that serve a catchment population of nearly 20 million people. AMPATH is the largest programme providing HIV care and treatment in Kenya; it has enrolled over 200,000 people living with HIV and currently provides antiretroviral therapy (ART) to over 127,000 patients [30] [25]. The selected facility is located at Moi Teaching and Referral Hospital (MTRH), the second largest referral hospital in Kenya, based in Eldoret. The AMPATH-MTRH clinic provides HIV care services for more than 45,000 PLHIV, approximately 33% of whom are aged ≥ 50 years. The Turbo Clinic is a rural health facility serving more than 10,000 PLHIV, 22% of whom are older adults, whereas Kitale Hospital, a peri-urban facility, provides HIV care services to more than 28,000 people, 17% of whom are older adults. The three facilities are equipped with an electronic tablet-based decision support interface called “point-of-care (POC)”, which allows healthcare providers to enter clinical visit information that is uploaded into the AMPATH medical records system (AMRS). At MTRH, noncommunicable disease (NCD) and HIV services are provided in separate buildings and by different providers. However, both Turbo and Kitale have integrated care for HIV and NCD within their HIV clinics.

Several community-based programs, including home-based HIV testing and counseling services, chronic disease screening and referral services using CHVs, have been successfully implemented by AMPATH in the catchment area [31–33]. Each CHV serves approximately 100 households within a defined geographical location, conducting home visits to provide basic health promotion and prevention services. Each community unit consists of 10 CHVs overseen by a community health extension worker.

#### Study population

We recruited the following groups for our project: OALWH and comorbid conditions, healthcare providers, CHVs, and older adults’ caregivers. Using purposive sampling, we aimed to capture a diverse range of perspectives from key stakeholders. For the older adult participants, a list of participants meeting the inclusion criteria of age 50 years or older, HIV-positive status, at least one comorbid condition, and active patient at the clinic was generated from the AMRS. Recruitment occurred during clinic visits to ensure representation across different conditions and included participants who lived at varying distances from the health facility. We requested that older adults refer their caregivers to our research assistants. Healthcare providers who had provided care for older people living with HIV and other comorbid conditions were purposively selected for participation.

AMPATH has established a longstanding relationship with communities in its catchment area, which we leveraged to identify and recruit community health volunteers. Collaborating with community leadership, we engaged local representatives, including village elders, religious leaders, and older adults themselves. Through the Trans Nzoia and Uasin Gishu County Directors of Health, we identified community units (consisting of 100 households) that allowed us to recruit CHVs heading these community units. We recruited CHVs from community units surrounding the three health facilities.

#### Data collection

All in-depth interviews (IDIs) and focus group discussions (FGDs) were conducted by three research assistants experienced in qualitative interview methods. Data were collected between November 2022 and March 2023. Table 1 shows the categories of the participants involved in this qualitative study.

##### IDIs

Research assistants utilized a semi structured IDI guide to conduct the interviews (Supplementary file 1). The guide was pretested for face validity with 2 OALWH—a male and a female—and the questions were revised to improve clarity. The guide included questions focusing on the challenges that OALWH face with respect to medication adherence, with a focus on individual barriers to and facilitators of medication adherence. A follow-up question was posed to gain a more in-depth understanding of their perceptions and thoughts about having a CHV visit their home to provide medication adherence support and to educate them on medication and general health. The interview sessions were held in a private room within the three health facilities. On average, the IDIs lasted between 45 and 60 minutes. IDI sessions were audio-recorded, and notes were taken by the research assistant for use during data analysis.

###### FGD:

We conducted six sex-disaggregated FGDs with OALWH. Prior studies [12] with OALWH in our setting have yielded limited open discussions among a mix of older women and men, hence our choice to separate FGDs for males and females. The FGDs with OALWH were used to triangulate the data and complement insights obtained from the IDIs. Six FGDs—three with healthcare providers and three with CHVs—assessed organizational and individual readiness to implement a community strategy for adherence support. An additional three FGDs with community members assessed contextual factors that may influence the implementation of the intervention, including their views on services offered by CHVs in the community and their perceptions of factors associated with medication adherence among older adults. The FGD guide (Supplementary file 1) was pilot tested for face validity with 10 individuals: OALWH (2 males, 2 females), 2 healthcare providers, 2 CHVs, and 2 community members; then, revisions to clarify the questions were made. In a confidential setting, trained moderators led the FGDs and facilitated the discussion, with a scribe taking notes. FGDs lasted between 90 and 120 minutes and were audio-recorded. To facilitate interactions between and among group members, the moderator ensured that each member had an opportunity to contribute. After each FGD, the moderator and the notetaker had a debrief session to summarize findings and compare notes. Participants were reimbursed for transport and served refreshments after the FGD.

### Data analysis

All audio recordings from our FGDs and IDIs were transcribed verbatim, and those in Kiswahili were translated into English by translators proficient in both languages. The data analysis followed an iterative approach consisting of four key stages. First, data familiarization was conducted through a thorough reading of the transcripts multiple times. Second, a coding framework was developed via NVivo 8.0, incorporating both deductive codes based on the consolidated framework for implementation research (CFIR)’s five domains (intervention characteristics, outer setting, inner setting, characteristics of the individuals, and the implementation process) and inductive codes emerging from the data [34]. A subset of transcripts, including two IDIs with OALWH and FGDs involving OALWH (2—one male, one female), CHVs (1), community members (1), and healthcare providers (1), was independently double-coded by the lead researcher (JK) and a research assistant at the AMPATH Qualitative Research Core. Any discrepancies in coding were resolved through discussion and consensus.

### Analytical framework

We adopted the CFIR as a guiding deductive analytical framework in our quest to identify the facilitators and barriers to implementing the proposed CBA, a patient-centered healthcare intervention [35]. Coded data were synthesized into CFIR domains and constructs, allowing for systematic comparisons among coders. Finally, the data were interpreted, methodologically synthesized, and compiled into the final report. The findings are structured within the CFIR, categorizing perceived facilitators and barriers into five key domains and mapped within 14 of the 39 CFIR constructs (Appendix 1).

## Results

We interviewed 166 participants, 84 (50.6%) females and 82 (49.4%) males, whose ages ranged between 25 and 80 years. The participant characteristics are summarized in Table 2.

### Perceived barriers to and facilitators of the implementation of community-based medication adherence support programs

Our findings are structured around the five domains of the CFIR framework described below and categorized within 14 of the 39 CFIR constructs shown in Table 3.

### Domain 1: Intervention characteristics

#### Perceived relative advantage and adaptability

##### Intervention is better than the current practice

our intervention, which entailed regular home visits whereby blood pressure measurements were taken and recorded, was perceived as effective and useful in providing immediate feedback on the effectiveness of medications and lifestyle changes while also reinforcing the importance of adherence. OALWH perceived that this intervention would reduce the cost of accessing blood pressure monitoring services, as this would be available to them from the comfort of their home. Healthcare providers view the collection and recording of quality BP data as a valuable tool for assessing and determining whether treatment adjustments are needed. They suggested that such regular monitoring could contribute to improved hypertension management.

“… because at times you are forced to go pay at the chemist to be checked (blood pressure measurement) if there is no hospital close by… so, if they will be checking it at home, it will be much better. It will be better because it will have saved on transport, saved on time, you will be able to take care of other things instead.”

CBA_IDI_OALWH_F_ELD (59 years)

The participants also perceived the use of teleconsultation as a tool that breaks geographical barriers and enhances access to healthcare services while improving patient–provider communication and providing timely support. OALWH felt that phone access to a clinician when needed would allow the patient to receive medical advice, consultations, and follow-ups without the need to travel, which was viewed as particularly beneficial for older adults with mobility issues or with transportation challenges.

“… I will also get to talk to the doctor if my (blood) pressure is not good since the health worker will give me the phone to talk to the doctor. It will really help those who are not able to get to the clinic because they are too old and need to travel long distances to get to the clinic”

CBA_IDI_OALWH_M_KTL_08 (62 years)

###### Modification to fit context:

While participants perceived the CBA intervention components as necessary, they felt that they could be tailored to patients depending on their needs. The frequency of visits to the patient’s home was viewed to be easily adaptable to suit the participant’s needs. For example, participants noted that not all patients will need teleconsultations with healthcare providers or medication refills by community health volunteers. One of the participants responded:

“Not everyone will need all the things that have been listed. For some people, home visits can be performed every two weeks; for others, visits can be monthly. There are also those who do not want to put all their medicines in the pillbox because they do not want anyone to see their ARVs, so those ones can be called to be reminded. I think the project can be flexible to allow one to choose what works best for them.”

CBA_FGD_CHV_TUR

The participants viewed the use of pill boxes as a simple way to reduce the cognitive load for those who struggled with remembering when and how to take multiple medications. They suggested that this could be modified to have simple instructions that minimize confusion and the risk of missing doses while offering healthcare providers a way to track medication use, as cited by a participant.

“… it will also assist us in pill count because we have clients who have high viral loads, and one of our interventions that we do is pill count. Therefore, some are so…they’ve mastered they know how many they should bring back. When you need two, they will always bring two. So, I think on that aspect also this arrangement will assist us.”

CBA_FGD_HCP_ELD

#### Cost

The participants viewed engaging CHVs stationed within the community to support medication adherence as a relatively cost-effective model that could be easily implemented. They noted that CHV home visits could reduce transportation costs for older adults who would otherwise need to travel to healthcare facilities for services such as blood pressure monitoring. However, CHVs expressed concerns that the intervention might increase their own expenses, particularly for traveling to assist older adults who require frequent check-ups and additional support.

“This will be costly for a CHV. If I have to visit an older patient regularly because they need close monitoring, it means I have to make more visits. The government is not even compensating enough to take a motorbike, and that means I will have to dig into my pocket and pay for the visits.”

CBA_FGD_CHV_KTL

“I will not need to travel to the facility for regular checkups for my blood pressure. I will not need to look for transport.”

CBA_IDI_OALWH_F_TRB (72 years)

#### Complexity

##### Multiple components of the intervention

The intervention, which includes home visits, medication organization, blood pressure monitoring, electronic data capture, and psychosocial support for OALWH provided by CHVs, was perceived as complex because of its multiple components. The participants frequently expressed concerns that complexity could lead to confusion among implementers. To address this, they recommended providing a clearer outline of CHV responsibilities and simplifying the intervention to focus on one or two key components for better feasibility and effectiveness.

“There are too many proposed components in the prototype, especially for a CHV. This seems to be complicated. It would be nice to make it simple, by maintaining easy things that the CHVs are already doing in the commuity and that will be easily continued even after the study.

CBA_FGD_HCP_ELD

### Domain 2: Process of implementing the intervention

#### Planning

##### Identification of the CHVs

During the planning phase of the intervention, participants emphasized the importance of selecting CHVs who are knowledgeable about medication adherence and, ideally, share the same HIV status as the participants. They suggested that CHVs with similar experiences could be more empathetic and provide effective support. OALWH indicated that shared experiences foster understanding and trust, enabling CHVs to better address their unique needs. One participant observed this

“For me, I would be comfortable with a CHV who is living with this disease (HIV) like me because I can discuss many things that we share in common whenever she visits, how they are managing it and laugh together. She must know about heart medicine too, so we can discuss more. I recently started using it myself. I have not had much experience.”

CBA_FGD_OALWH_KTL

#### Engaging

##### Capacity building needs of CHVs

Participants emphasized the importance of training CHVs in key areas such as medication adherence, motivational interviewing, data collection, and blood pressure measurement to equip them with the necessary skills to effectively support older adults in the community. They also highlighted the need to provide CHVs with essential tools, including blood pressure machines, adequate batteries, and pillboxes, to ensure that they can perform their roles efficiently.

“This can be truly great to help continue care to the community and, in a way, help get more information about the patient. If the CHVs are trained on antihypertensives, including common side effects, they can help identify any issues early. Our clients always wait until they return to the clinic for us to know what happened if they still remember. Therefore, this is nice. …… Additionally, since PIC4C (a project that implemented an NCD screening at the community using CHVs) trained most of them to conduct screening, they should be competent with BP measurements and data collection. may be just a refresher”

CBA_FGD_HCP_KTL

#### Executing

##### Supportive Supervision

Participants highlighted the critical role of supportive supervision—an approach that emphasizes mentorship, collaboration, and continuous learning for CHVs—as a facilitator of the successful implementation of CBA intervention. Regular supervision, the availability of HCPs for consultations, and ongoing education to address areas where CHVs need clarification were viewed as essential to enhancing CHVs’ knowledge and confidence in supporting OALWH.

“CHVs will need to be supported regularly by someone going to the field to ensure they are confident in doing what they are meant to do, say taking BP measurements… Additionally, if they have a go to person in the facility, that they can always call to get one or two clarifications, it will truly improve this project”

CBA_FGD_HCP_ELD

##### Lack of a clear referral protocol

Participants were concerned about the referral process for patients needing advanced clinical care by specialists or emergency hospitalization. They identified a poorly designed referral process that lacks clarity on the role of CHVs in the process and a lack of clear referral protocols as a barrier to the effectiveness of the intervention. This lack of clarity was cited as a challenge for CHVs in ensuring that critical cases receive timely and appropriate care.

“I may go to the household and find that the person I am visiting is very sick or is now just weak and sleeping in the house. Maybe they have not even eaten, let alone taken their medicines. If the clinician says that we refer this person to the facility, knowing where to tell them to go if not specified by the clinician can be difficult. Then, sometimes they do not have transport or means to reach the hospital. Now, the family will think it is my responsibility to take them to the hospital, and I have my own struggles. This can be very challenging.”

CBA_FGD_CHV_TRB

### Domain 3: Outer setting

#### Patient needs and resources

The participants identified patients’ unmet needs as potential barriers to implementing the proposed intervention. The needs that were mentioned included social support, food, and money to cover the costs of NCD medication or purchase medical insurance. In addition, participants perceived having treatment supporters other than CHVs, such as family members or friends, to be an important component of the intervention. The need for older adults’ caregivers as additional support to patients was cited as key to enhancing medication adherence among OALWH, with the proposal of incorporating them into interventions for sustainability.

“Since the client is taking medication, they require food. We will go and see how they are doing, but probably the person has no supper. Since morning, they have not taken breakfast and have to take medications such as five tablets…. Money is another need. They may not have money to buy medicines for hypertension….”

CBA_FDG_CHV_KTL

“… I think in addition to CHVs, the treatment supporters in the household should also be engaged. That way, even if the CHV is not around, they are able to arrange medication for the patient.”

CBA_FGD_CM_ELD

#### External policies and incentives

##### Policies and incentives for CHVs

Participants perceived the stipend received by CHVs from the Kenyan government as a factor that may limit visits to older adults’ households for medication adherence support. The logistical constraints faced by CHVs, such as incurring personal transport costs in reaching patients, particularly in remote areas, were cited as an impediment to intervention implementation. The participants suggested the reimbursement of personal costs incurred to implement the intervention to mitigate against CHVs using personal funds to supplement project activities.

“The government remunerates them (CHVs), but 2000 shillings a month is so little to cover frequent visits.”

CBA_FGD_HCP_ELD

##### Health financing

Participants also identified the lack of dedicated funding for chronic diseases, in comparison with the free provision of HIV services, as a significant barrier to implementing the CBA intervention. Specifically, they mentioned challenges such as medication stockouts and the inability of patients to afford treatment. Enrolling patients in the National Health Insurance Fund (NHIF) was perceived as a key strategy to reduce out-of-pocket expenses for hypertension medications. By covering a substantial portion of medication costs, the NHIF was seen as an enabling factor for older adults to adhere more consistently to their prescribed treatment regimens, making care more financially accessible.

“Drugs for NCDs are not free. One has to buy them. If you don’t have NHIF, you pay out of pocket. If the issue of stock outs for NCD medication is not sorted, this is going to affect medication adherence.”

CBA_FGD_HCP_KTL

### Domain 4: Inner setting

#### Mission alignment

##### Patient-centered care –

Participants perceived the delivery of the intervention to be in line with the needs of OALWH. Healthcare providers shared their aspirations of offering patient-centered care and perceived interventions that allowed coordinated care delivery to reduce missed appointments among patients while ensuring regular follow-ups and increased adherence to medication. OALWH alluded that a reliable supply of NCD medications in the facility and minimizing supply chain disruptions were crucial to the successful implementation of CBA intervention.

“Clinic visits for all the services for the patient can be scheduled for one day so that they don’t have to come to the hospital multiple times. That way, it will be helpful even for the CHV to remind the patient on when to come to the clinic next”

CBA_FGD_CHP_TRB

#### Team culture

##### Enhanced collaboration between CHVs and healthcare providers

Participants indicated that devising a clear channel of communication and coordination will ensure that all members of the healthcare team are aligned in their approach to the care of the patient. The alignment helps create a seamless care continuum where CHVs can relay important patient information to HCPs and vice versa. This, they said, would ensure that any issues related to medication adherence are promptly addressed. In addition, the supportive supervision of CHVs through training and mentorship by facility HCPs will increase their capacity and enhance their skills in managing medication adherence.

“…the doctors at the facility will help us with continuous learning. Let us say, I am not sure of something; I can call him/her and will get clarification. This will truly help a lot.”

CBA_FGD_CHV_KTL

##### Poor Communication with Providers

Participants raised concerns about inadequate instructions provided by healthcare workers, which they felt could result in incomplete, incorrect, or conflicting information about medications. Older adults expressed that the information given by HCPs is often confusing or difficult to understand, potentially affecting proper treatment dosing. One participant observed the following

“They don’t give you time to explain. Therefore, they tell you, ‘Go divide this drug into two and take one in the evening and another the next day.’ They don’t explain very well. You can go home not having clear information on how to do things.”

CBA_FGD_OALWH_KTL

### Domain 5: Characteristics of the individuals involved

#### Beliefs

##### Low CVD risk perception

Participants expressed concerns that individuals with a low-risk perception of cardiovascular disease would not fully appreciate the importance of adhering to prescribed medications. This underestimation of health risks could lead to complacency, reducing their willingness to engage with CHVs or accept home-based support. One participant illustrated this challenge

“I know some people who can truly benefit from this support. However, even visiting them can be difficult because they believe that they will not experience stroke or other conditions that are likely to occur if they do not take the medicines. They tell you, I am taking my medicine well; I don’t need someone to remind me, but when you check their viral load, it is not going down. Here you know this is a problematic patient.”

CBA_FGD_CHP_TRB

#### Knowledge

##### Low levels of knowledge on medication for hypertension

Participants were concerned that the low levels of knowledge about hypertension medication among OALWH and CHVs would lead to inaccurate dosing and timing of medication intake. They were concerned that when CHVs do not feel adequately informed, they may struggle to correctly educate patients, address concerns, or manage complex medication schedules, which may lead to misinformation, confusion, and ultimately, poor adherence to prescribed regimens. A participant expressed this concern

“I don’t know the drugs that people living with hypertension are given and how they are taken. Sometimes drugs may have certain effects that I cannot explain or even relate it to that drug. In truth, the person you are going to visit may even know more about the medicine than you do and can’t even help them after all. If I have to do this, I will truly need to be educated and ensure I have someone I can consult if I find something that is challenging.’

CBA_FGD_CHV_ELD

##### Declining cognitive ability

Participants reported that declining neurocognitive function among OALWH makes it increasingly challenging for them to understand complex medication regimens and instructions. Healthcare providers expressed concerns that many OALWH might struggle to remember to take their medications consistently, potentially resulting in missed doses or incorrect administration, even when CHVs assist with medication organization at home. Furthermore, the growing burden of chronic comorbidities among older adults was seen as a significant challenge to implementing the intervention. Managing multiple conditions often involves complex medication regimens with varying schedules and instructions, which participants perceived as overwhelming for some patients, increasing the risk of confusion and nonadherence. A participant highlighted this concern

“Older patients forget things very fast. We encourage them to come with someone to the clinic so that we can give them instructions on drugs and how and when to take them. Although the CHV will help in reminding them on adherence, they might still forget, as CHVs will not be present every day to perform direct observed therapy.”

CBA_FGD_HCP_TRB

#### Personal attributes

##### Trust between CHVs and older patients

Participants reported that when older adults trust CHVs, they are more likely to engage openly in discussions about their health, share concerns, and ask questions about their medications. This open communication was perceived to be vital in identifying barriers to adherence and tailoring support for OALWH. Additionally, participants indicated that open communication may foster a proactive approach to patients’ management of their health and prescriptions, as exemplified by this participant

“I want someone I can trust to come to my home so that I can talk to them freely. Someone who is patient with me, and I can share my personal information. I do not want someone who will go and gossip about me, and they are not helping to solve my issues.”

CBA_IDI_OALWH_F_KTL_05 (61 years)

##### Empathy

Participants emphasized that engaging with CHVs provides a valuable opportunity to offer psychological support to OALWH, particularly those living alone. This empathetic support was seen as instrumental in helping patients cope with the emotional stress and challenges associated with managing chronic diseases, fostering a sense of connection and understanding in their care journey. A participant observed as follows

“It will be of benefit because, like I am telling you, there are people who are living lonely lives. They feel like they have been neglected because they are sick. But if you have someone who feels for you, comes to encourage you and tell you that there is still life, and life will still continue, you see with that even if you were losing hope you will start to gain faith slowly by slowly.”

CHV_IDI_OALWH_M_ELD_02 (77 years)

## Discussion

This formative study explored the perceptions of key stakeholders in the possible implementation of a CHV-led medication adherence support program (CBA) for older adults living with HIV and hypertension. Overall, our findings revealed that, when examined through the CFIR domains, CBA holds promise for improving health outcomes, particularly for people living with HIV and managing other chronic conditions. Leveraging the facilitators, which included the perception that CBA intervention is better than the current practice, its adaptability, a well- laid out implementation plan, and trust among CBA stakeholders while mitigating barriers such as the potential increased cost of implementation and the complexity of the intervention, in addition to the low levels of hypertension knowledge, CBA has the potential to promote medication adherence and contribute to the improved health outcomes of older adults living with HIV and hypertension.

The intervention characteristics domain, which emphasizes the importance of tailoring interventions to align with the specific context, was identified as a pivotal factor in the successful implementation of CBA. Both the adaptability and the relative advantage of the intervention were identified as facilitators, whereas the complexity and costs emerged as significant barriers to the effective delivery of the CHV-led intervention. A potential barrier to CBA intervention is its complexity, which is due to its multiple components. Research on intervention characteristics suggests that simpler interventions are more effective, as they improve user satisfaction and enable quicker mastery of their components [36]. Therefore, simplifying CBA intervention components and establishing clear, standardized guidelines could enhance usability and promote fidelity to the intervention [37, 38]. On the positive side, our study highlighted that CBA is better than the current practice is, with the potential for tailoring it to meet the needs of older adults, a factor that has been documented to increase the adoption of an intervention [39]. The integration of electronic data capture within the intervention provides the opportunity for immediate feedback on medication effectiveness, thereby supporting better management of hypertension. The use of pill boxes was also recognized as a simple yet effective tool for reducing cognitive load, helping patients remember to take their medications correctly. Additionally, teleconsultations are perceived as valuable for improving access to healthcare services and enhancing communication between patients and providers while overcoming distance barriers [40, 41]. These facilitators, coupled with the development of standard operating procedures, could simplify the intervention and improve its adoption. Similar to our findings, other studies have shown that engaging CHVs and offering short training programs can be a cost-effective and sustainable model, leveraging their familiarity with the community to support medication adherence for OALWH [42, 43]. However, to ensure the long-term sustainability of the intervention, compensating CHVs at a rate beyond government-established guidelines is necessary, as documented in other studies utilizing CHVs to implement interventions in SSA [44–46].

In our study, the implementation process domain was recognized as a critical factor in the successful planning and execution of the intervention. A potential barrier identified by participants was the lack of clear referral protocols that would facilitate older adults needing this support. This issue aligns with findings from other studies, which emphasize the importance of clear guidelines in the implementation of interventions to improve fidelity [37, 38]. Dysfunctional referral systems, coupled with delays in accessing facilities, can result in a lack of timely intervention and poor health outcomes, as highlighted in previous research on community-based health interventions [47]. Despite this barrier, our study identified several facilitators within the planning, engaging, and execution phases of the intervention. The process of carefully selecting CHVs who are familiar with the community context was recognized as essential for improving engagement and trust with patients. Capacity-building initiatives, such as training CHVs on medication adherence and the provision of ongoing supportive supervision, were also key to the successful implementation of the intervention. These findings are consistent with the literature, which suggests that well-trained and supported CHVs are more effective at delivering healthcare interventions, as they are better equipped to address the diverse needs of patients and ensure adherence to treatment regimens [48, 49]. By addressing the identified barriers and enhancing the capacities of CHVs through training and supervision, the intervention’s sustainability and impact can be significantly improved.

Within the outer and inner setting domains of the CFIR framework, our study revealed several barriers and facilitators impacting the success of the intervention. Unmet patient needs, such as a lack of social support and basic necessities, were identified as key challenges, which aligns with the literature suggesting that socioeconomic factors significantly influence the effectiveness of health interventions, particularly for older adults managing multiple chronic conditions [50]. Limited health financing for noncommunicable disease (NCD) medications was an additional barrier that hindered the intervention’s success. Studies in SSA have revealed a relative lack of financial protection for access to medications for managing NCDs [51]. Furthermore, poor communication and increased workload for CHVs were also identified as barriers, echoing findings from other studies that highlight how strained resources and ineffective communication can compromise healthcare delivery [49].

In a fragmented care system, proper scheduling and follow-up systems can help manage the complex healthcare needs of OALWH, making it easier for them to adhere to their treatments. Integrated care models that combine HIV and NCD management provide a holistic approach to patient health [52, 53], a factor that was perceived as key to the successful implementation of CBA intervention. On the other hand, leveraging facilitators that include enhanced collaboration among stakeholders, the engagement of treatment supporters, and a mission to provide patient-centered care, all of which have been documented in previous research as strategies that improve the implementation and sustainability of health interventions [54, 55], may optimize the delivery of care and medication adherence support for OALWH.

Within the characteristics of the individuals engaged in the intervention domain, our study identified low levels of medication knowledge, declining cognitive ability, and treatment preferences due to side effects as barriers to implementing the intervention. Patients with a limited understanding of medication purposes and regimens, coupled with low cardiovascular disease risk perception, have been associated with poor adherence to hypertension medication [56]. Enhancing patients’ and medication supporters’ education and knowledge of potential complications as a result of nonadherence is essential for improving adherence. The literature suggests that modifying interventions to account for cognitive impairments by incorporating simplified instructions, instituting reminders, and providing caregiver support, such as CHVs, enhances adherence [57, 58]. However, trust between CHVs and older patients and empathy by CHVs were identified as vital facilitators that may foster the open communication and empowerment of OALWH. When CHVs approach patients with genuine empathy and understanding, it creates a safe and supportive environment where OALWH feel more comfortable disclosing personal health information and discussing challenges they may face in adhering to their medication regimen [48]. This emotional connection can enhance patient–provider relationships and empower OALWH to actively participate in their care, ultimately improving treatment adherence and health outcomes [59]. Empathetic interactions also build trust, encouraging OALWH to seek help and follow medical advice, knowing that their concerns will be listened to and addressed respectfully.

### Study strengths and limitations

This qualitative study included diverse voices captured through in-depth interviews and focus group discussions with OALWH, healthcare providers, CHVs, and community members, allowing for an in-depth exploration of contextual factors that may influence community-based medication adherence support interventions for older adults. Combining in-depth interviews with focus group discussions allowed for the triangulation of data, enhancing the credibility and validity of the findings. The application of the CFIR framework allowed for a systematic identification of both facilitators and barriers across the five key domains. This comprehensive framework ensured that our analysis was grounded in a well-established theoretical model, enhancing the rigor and generalizability of our findings. However, the study’s participant selection process may have introduced selection bias. While efforts were made to ensure a diverse sample, the purposive sample, reliance on the AMPATH networks within the community and recruitment during clinic visits may have resulted in the inclusion of participants who are more motivated or have stronger opinions about the CHV-led intervention, potentially skewing the results.

## Conclusion

Our study sheds light on the factors that may influence the implementation of patient-tailored community-based medication adherence support programs for OALWH, particularly in the context of emerging challenges associated with comorbidities and complex chronic disease medication regimens. The identified facilitators and barriers within CIFR highlight the complexities of tailoring community support services to the needs of patients. By acknowledging and mitigating the identified barriers while leveraging facilitators, healthcare systems can strive for more patient-centered, community-driven strategies that are not only acceptable but also more sustainable in enhancing medication adherence in older adults living with HIV and comorbidities.

## Supplementary Files

This is a list of supplementary files associated with this preprint. Click to download.

- Appendix1AnalysisCode.docx

## Figures and Tables

**Table 1:** Categories of participants in FGDs and IDIs

Data collection method	Participants	Number of participants	Number of participants per Facility
**FGDs**	OALWH with chronic comorbid conditions	56	MTRH – 20
			Turbo – 17
			Kitale – 19
	Community health volunteers	25	MTRH – 9
			Turbo – 8
			Kitale – 8
	Healthcare providers	27	MTRH – 10
			Turbo – 8
			Kitale- 9
	Community members (8 village elders, 5 religious leaders, 8 caregivers of older adults and 7 older adults)	28	MTRH – 9
			Turbo – 9
			Kitale – 10
**IDIs**	OALWH with chronic comorbid conditions	30	MTRH – 10
			Turbo – 10
			Kitale – 10

**Table 2:** Characteristics of the study participants

Categories of Participants		Number of participants	Female	Male
OALWH		56	31	25
Community health volunteers		25	11	14
Healthcare providers	Clinical Officers	18	8	10
	Nurses	9	6	3
Community Members	Village elders	8	2	6
	Religious leaders	5	2	3
	Caregivers	8	6	2
	Older adults	7	3	4
OALWH with chronic comorbid conditions		30	15	15

**Table 3:** Perceived facilitators of and barriers to the implementation of CBA interventions categorized within CFIR domains and constructs

CIFR Domain	Constructs	Factors influencing the implementation of CBA intervention	
		*Facilitator*	*Barrier*
Intervention characteristics	Relative advantage and adaptability	Better than the current practice Can be modified to fit the context	
	Complexity		Multiple components of the intervention
	Cost		Increased cost for CHVs
Process of implementing the intervention	Planning	Identification of the CHVs	
	Engaging	Capacity building for CHVs	
	Executing	Supportive supervision	Lack of clear referral protocol
Outer setting	Patient needs and resources	Engagement of treatment supporter	Patient’s basic needs
	External policies and incentives		Policies and incentives for CHVs Healthcare financing
Inner setting	Team Culture	Enhanced collaboration	Poor communication
	Mission alignment	Patient-centered care	
Characteristics of individuals involved	Beliefs		Low CVD risk perception
	Knowledge		Low levels of knowledge on hypertension medications Declining cognitive ability in older adults
	Other Personal attributes	Trust between CHVs and older adults Empathy	

## Data Availability

Data from this study contain information that may compromise the confidentiality and privacy of the participants. However, requests made by the corresponding author are considered and available as appropriate. Requests can be submitted to the corresponding author at chiri2809@gmail.com

## Abbreviations

AMPATH: Academic model providing access to healthcare
AMRS: AMPATH medical records system
CBA: community–based medication adherence support
CFIR: Consolidated framework for implementation research
CHVs: Community health volunteers
CVD: cardiovascular disease
ELD: Eldoret
FGD: Focused group discussion
HCP: Healthcare providers
IDI: in–depth interviews
KTL: Kitale
MTRH: Moi Teaching and Referral Hospital
NCDs: noncommunicable diseases
NHIF: National Hospital Insurance Fund
OALWH: older adults living with HIV
PLHIV: People living with HIV
POC: point of care
SSA: Sub–Saharan Africa
TUR: Turbo
